# Ultra‐fast dosimetric data collection with a commercial plastic scintillation detector in an MR‐linac

**DOI:** 10.1002/acm2.70460

**Published:** 2026-01-07

**Authors:** Carlos Ferrer, Concepción Huertas, Marcos Feijoo, Alessandro Cardin, Moisés Sáez

**Affiliations:** ^1^ Department of Medical Physics and Radiation Protection La Paz University Hospital, Paseo de la Castellana 261 Madrid Spain; ^2^ Blue Physics LLC, 19145 long lake ranch blvd Lutz Florida USA

**Keywords:** commissioning, MR‐linac, plastic scintillation detector, Ultra‐fast

## Abstract

**Background:**

Plastic scintillation detectors (PSD) are widely used for detecting and measuring ionizing radiation. These detectors are versatile, with high efficiency, fast response and the ability to provide real‐time measurements.

**Purpose:**

Evaluate the suitability of Blue Physics PSD (BP‐PSD) for performing ultra‐fast dosimetric commissioning measurements with high accuracy and precision in a very short time.

**Methods:**

Ultra‐fast measurements were performed in water using a BP‐PSD on an Elekta Unity MR‐linac. Percentage depth doses (PDD) and profiles at different depths were measured at two movement velocities, 10 mm/s and 20 mm/s, for field sizes ranging from 10 × 10 cm^2^ to 1 × 1 cm^2^. Gamma analysis was conducted to compare these measurements with those obtained during machine commissioning using a PTW Semiflex 3D ionization chamber (for PDD) and a PTW micro‐Diamond detector (for PDD and profiles). Gamma criteria of 2%/2 mm and 1%/1 mm dose difference/distance to agreement were studied, alongside field size, penumbra, and measurement time.

**Results:**

All PDD and profile gamma passing rates were 100% at 2%/2 mm. At the stricter 1%/1 mm criteria, all PDD showed a passing rate above 96.97% for both velocities, with most of the profiles exceeding 95% at 10 mm/s and 90% at 20 mm/s. Gamma analysis results were superior for smaller fields (1 × 1 cm^2^ and 2 × 2 cm^2^) and generally better at 10 mm/s. On average, the penumbra measurements obtained with the PSD were greater than those achieved with the micro‐Diamond detector. Measurement times were found to be between 7 and 14 times shorter for PDD, and between 5 and 9 times shorter for profiles at speeds of 10 mm/s and 20 mm/s, respectively.

**Conclusions:**

Ultra‐fast measurements using the Blue Physics PSD are suitable for acquiring dosimetric commissioning data with high accuracy and precision, and can be performed in a much shorter timeframe than with commonly used detectors.

## 1 INTRODUCTION

The installation of new radiotherapy equipment in a radiation oncology department, such as a linear accelerator (linac), always involves two distinct processes: acceptance and commissioning. During acceptance, the vendor demonstrates the system's performance against dosimetric and mechanical specifications, as well as its overall functionality. Following acceptance, the dosimetric commissioning process establishes the treatment beam characteristics required for clinical application. The data collected during commissioning process serves as a reference for future dosimetric quality assurance (QA) procedures and for treatment planning system (TPS) modelling.^1^

The dosimetric commissioning of a linac usually entails several of steps, including the measurement of percentage depth doses (PDD) and dose profiles across a range of field sizes and depths. These measurements are performed in accordance with the requirements of the TPS algorithm.^2^, ^3^, ^4^, ^5^ Typically, they are carried out in water phantoms using ionization chambers (IC) and diode detectors. The selection of appropriate detectors and measurement techniques must be made with care, in line with established professional guidelines.^5^, ^6^ Particular attention must be given to small field sizes (≤3 × 3 cm^2^), for which extended sampling times are required to improve the signal‐to‐noise ratio.^5^, ^7^

In addition to IC and diode detectors, plastic scintillation detectors (PSD) have been proposed as suitable instruments for beam data commissioning.^8^, ^9^ These detectors are nearly temperature‐independent, exhibit less radiation damage than diodes, and better energy dependence than other detector types. Moreover, they are water equivalent, resulting in a $k_{Q_{\mathrm{clin}},Q_{\mathrm{msr}}}^{f_{\mathrm{clin}},f_{\mathrm{msr}}}$ value close to unity. They can also be manufactured with small dimensions, making them appropriate for use in small field dosimetry.^8^, ^10^, ^11^, ^12^

Another key attribute of PSDs is their time resolution. The temporal characteristics of an organic scintillator depend on the scintillating material, as well as the type of scintillator, the nature of the ionizing particles and the energy of the incident radiation. Organic scintillators typically reach their peak response within approximately 10^−^^9^ s, with signal decay occurring over 10^−^^8^ to 10^−^^9^ s.^12^, ^13^

In the field of radiotherapy, PSDs have been utilized for a range of applications^13^, ^14^ including in vivo dose verification in brachytherapy^15^, ^16^ photon external beam radiotherapy (EBRT)^10^, ^17^, ^18^ and proton therapy, predominantly in the form of scintillating sheets^19^, ^20^, ^21^, ^22^, ^23^ or arrays^24^, ^25^ Previous works with scintillators have reported excellent results for patient‐specific QA of VMAT SRS treatments.^26^, ^27^ Plastic scintillators have also been used in ultra‐high dose rate as in proton FLASH radiotherapy^23^, ^28^, ^29^ and in innovative systems combining positron emission tomography (PET) with a linac for biology‐guided radiotherapy (BgRT).^30^

Data acquisition during commissioning for TPS modelling and subsequent machine QA is a complex and time‐consuming task for medical physicists, and can take up to four weeks or more.^3^, ^31^ Various approaches have been proposed to reduce this time. One such strategy involves the use of ‘golden’ datasets has been proposed as a potential solution. Several studies have reported that site‐specific commissioning data are consistent within 1% of manufacturer‐provided or previously published datasets for identical linac models.^32^, ^33^, ^34^ However, the American Association of Physicists in Medicine (AAPM) has cautioned that such datasets should be used judiciously, given the potential lack of reproducibility between individual linacs.^5^, ^35^ In addition, artificial intelligence (AI) and machine learning techniques have been investigated for dosimetric data verification and for reducing the number of measurements required^36^ as well as neural representation learning.^37^ Nonetheless, these approaches necessitate large volumes of training data and carry a risk of oversimplifying the variability inherent in clinical systems.

The commissioning of a magnetic resonance image‐guided linac (MR‐linac), which integrates a linac with a magnetic resonance imaging (MRI), also involves PDD and dose profile measurements for beam characterization.^38^, ^39^, ^40^, ^41^ It should be noted that dosimetry in a magnetic field is challenging due to the need to account for the magnetic field effect on secondary electrons, which is caused by the Lorentz force.^42^, ^43^

The objective of this study is to evaluate the potential of a commercial PSD, characterized by fast temporal response and high spatial resolution, to significantly reduce the time required for dosimetric commissioning of an MR‐linac. The proposed approach enables ultra‐fast, accurate, and precise data acquisition while maintaining conventional beam data collection methodologies.

## 2 METHODS

Measurements of PDD and dose profiles for beam characterization were conducted on an Elekta Unity MR‐linac with 7 MV flattening filter‐free (FFF) nominal photon energy and with a 1.5 T magnetic field. A detailed description of the Elekta Unity system (Elekta AB, Stockholm, Sweden) can be found in the literature.^43^, ^44^, ^45^, ^46^ The source axis distance (SAD) of the system is 143.5 cm, with the treatment couch surface located 14 cm below isocenter.

Ultra‐fast measurements were carried out using the Blue Physics (BP) Model 10 PSD (Blue Physics LLC, Lutz, FL, USA) placed in a BEAMSCAN MR water phantom (PTW, Freiburg, Germany). This PSD has been described and characterized in previous studies.^47^, ^48^, ^49^ The Blue Physics PSD (BP‐PSD) consists of a cylindrical polystyrene plastic scintillator, 1 mm in diameter and 1 mm in length, with a sensitivity volume of 0.785 mm^3^, embedded in an acrylic cladding material. Its principal advantage for ultra‐fast measurements lies in its response time. The BP system readout time is 10 µs and can sample at 104 kHz (700 µs)^47^, ^48^, ^49^ while the BP software provides real‐time graphical display of individual pulses emitted by the linac. Given these characteristics, it was proposed to name this measurement method “ultra‐fast.”

### 2.1 Water phantom set up

The PTW BEAMSCAN MR is a motorized water phantom designed for beam data acquisition in magnetic fields up to 1.5 T. It features a scanning range of 568 mm x 145 mm x 355 mm, with patented TRUFIX^BS^ system for axial and radial detector setup The phantom comprises a PMMA tank and a wheeled trolley acting as a water reservoir. It is operated via the PTW BEAMSCAN software package (v.4.4), which includes several independent modules controlled by Mephysto Navigator software. This software allows the detector to be positioned and all the measurement parameters to be selected, such as range, number of measurement points, speed of movement between them and measurement time at each point. It is important to note that increasing the measurement time at each point improves the signal‐to‐noise ratio (SNR) and reduces uncertainty, which is particularly valuable for small field measurements.^50^, ^51^ This water phantom supports point‐by‐point acquisition using IC, diode detectors, or micro‐Diamond detectors. However, it is not possible to take measurements in continuous mode by design, limiting rapid acquisition capabilities. Furthermore, the water phantom has the capacity to accommodate two detectors, which are necessary to measure field sizes larger than 10 × 10 cm^2^. This issue is due to the dimensions of the rail on which the detectors are mounted. The rail is not long enough to cover the entire 568 mm field width in the cross‐line direction. PTW software utilises two detectors to obtain a profile that covers the entire cross‐line direction for fields greater than 10 × 10 cm^2^. Consequently, as with BP‐PSD only one scintillation detector is allocated, the 10 × 10 cm^2^ field size was the largest measured in this study.

The PMMA tank was positioned on the MR‐linac couch and the first alignment procedure was performed. It should be noted that Elekta Unity does not have lasers or field light to position the water phantom. The procedure involves the placement of a plastic base plate with three radiopaque ball bearings into the empty tank. An MV image was acquired and analyzed with Elekta software to verify the correct positioning of the phantom relative to the radiation field. If tank rotation was identified, correction was applied by placing stepped plastic shims on either side of the +X end of the tank. No additional alignment adjustments were made during ultra‐fast measurements.

### 2.2 Measurements

#### 2.2.1 BP‐PSD setup

Two systems were utilized to perform the ultra‐fast measurements with the BP‐PSD detector. Mephysto software was used solely for controlling the tank water filling and the initial centering of the detector. The centering, position, movement speed and signal reading of the BP‐PSD were controlled using the BP electrometer and software. The software controls the mechanism connected to the tank and obtains dosimetric data via an Ethernet connection with the water phantom.

**FIGURE 1:**
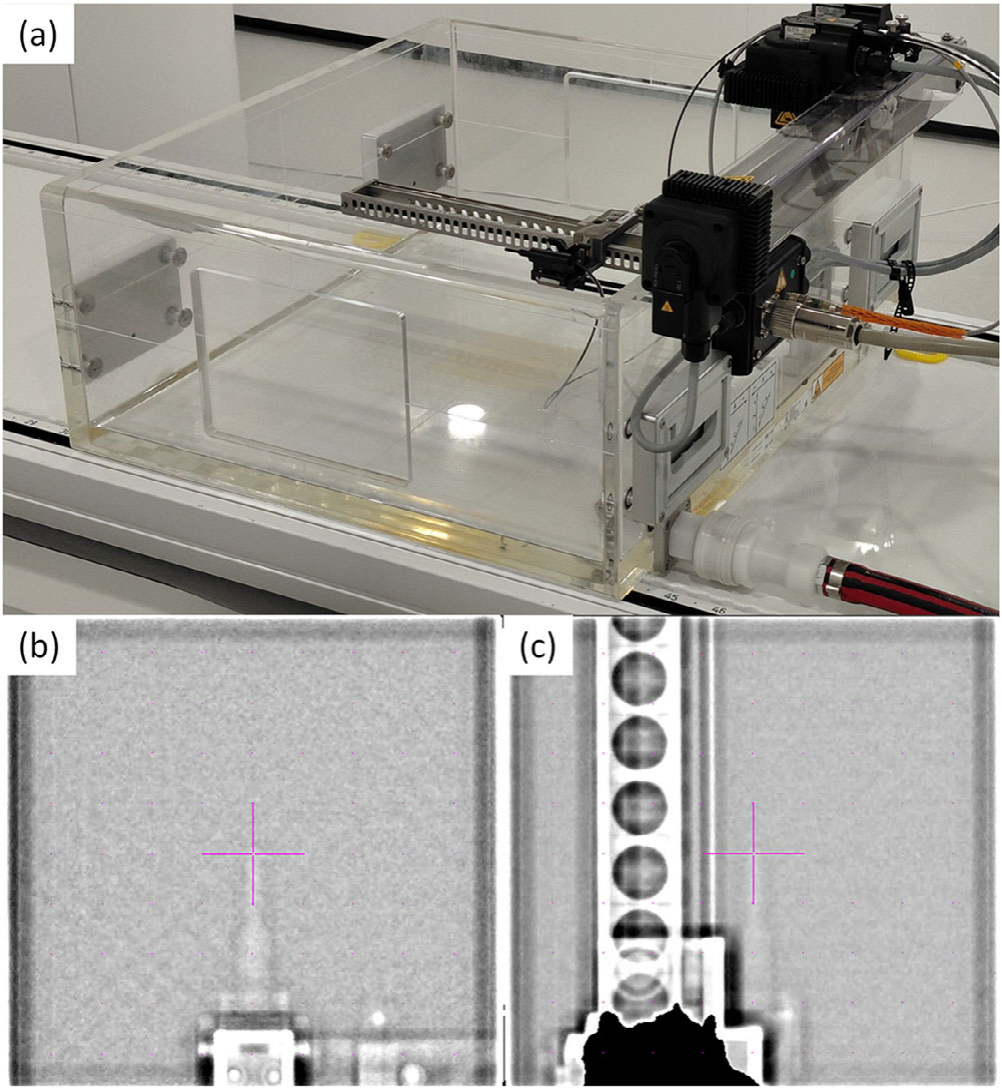
(a) BP‐PSD detector placed in the water phantom. MV image of the BP‐PSD placed at the isocenter (marked with red cross) at gantry angles (b) 0° and (c) 270°.

The BP‐PSD was mounted in the tank using a PTW T21008.1.150 Trufix ^BS^ detector holder and T4316/U6002 stop thimble designed for Semiflex 3D TW31021 IC, with the detector stem aligned perpendicularly to the beam axis as shown in Figure 1a. A preliminary centering of the BP‐PSD was conducted with Mephysto software, which is used to place the BP‐PSD at the mechanical isocenter with the assistance of the Unity MV imaging subsystem, which acquires images with an MV detector panel placed opposite to the beam target. This was done in the absence of water, since the BP‐PSD is water‐equivalent and cannot be discerned with MV images once the water tank is filled (Figure 1b,c). Once the BP‐PSD was initially centered, the PMMA tank was filled with water until the mechanical isocenter remained 10 cm below the water surface. The source‐to‐surface distance (SSD) was found to be 133.5 cm, consistent with the standard commissioning setup.

Prior to the ultra‐fast measurements, the BP‐PSD was calibrated in accordance with the method described by Underwood *et al* ^52^ to separate the primary scintillation signal from that generated by the Cerenkov effect. Calibration was performed using 10 × 10 cm^2^ and 4 × 4 cm^2^ square fields. The BP software displays the measured pulses emitted by the linac graphically in real‐time and provides the results by subtracting the Cerenkov from the sensor reading^47^, ^48^ and delivers both values.

With the BP‐PSD placed at the mechanical isocenter, the radiological isocenter is set using the BP software in accordance with the AAPM TRS‐483 recommendations.^7^ The software assumes control of the water phantom and, hence, of the BP‐PSD movement. The radiation was delivered for a 5 × 5 cm^2^ square field size and the BP software searched the radiological isocenter in an automated task moving the BP‐PSD towards the four sides of the radiation field. After locating the radiological center, the BP‐PSD was finally centered and placed on the water surface. This location was designated as the origin of the measurements.

#### 2.2.2 Micro‐Diamond diode and Semiflex 3D measurements setup

Point‐by‐point measurements were performed during the machine commissioning process using the PTW software. The PTW Semiflex 3D TW31021 IC with an active volume of 0.07 cm^3^ and the PTW micro‐Diamond TW60019 detector with an active volume of 0.004 mm^3^ were utilized to acquire PDD and profiles. The standard procedure with the water phantom in the presence of the magnetic field was followed. PDD were measured in 5 mm increments at depths between 130 and 120 mm, in 2.5 mm increments at depths between 120 and 25 mm, and in 1 mm increments at depths between 25 mm and the water surface. Movement speed between points was 10 mm/s, with 0.25 s measurement time per point. The measurement of profiles was conducted at a constant velocity of 10 mm/s between designated points, with a step size of 2.5 millimeters. However, in the penumbra region, the step size was reduced to 1 millimeter. These data were utilized in this study for the purpose of comparison with BP‐PSD measurements.

#### 2.2.3 Ultra‐fast measurements

Ultra‐fast measurements were acquired using only the BP software and electrometer. The BP electrometer, connected to the water phantom, controlled both scanning speed and detector positioning, while the software displayed the results. Measurements were performed for field sizes of 1 × 1, 2 × 2, 3 × 3, 5 × 5 and 10 × 10 cm^2^ at scanning speeds of 10 and 20 mm/s to assess any speed‐related effects. PDD were measured at the beam axis, from the water surface to a depth of 13 cm, and normalized to the maximum dose. To evaluate beam declination with depth, two profile 5 × 5 cm^2^ field size measurements were taken on the beam axis and at different depths, so that the software can correct for it. In‐plane (Y) and cross‐plane (X) profiles were measured at 1.3 (d_max_), 5 and 10 cm (isocenter) depth for each field size (Table 1).

**TABLE 1:** Measured field sizes, depths, and speeds.

Field size (cm^2^)	Speeds (mm/s)	Profile depth (mm)
1 × 1	10	13
2 × 2	20	50
3 × 3		100
5 × 5		
10 × 10		

### 2.3 Data analysis

**FIGURE 2:**
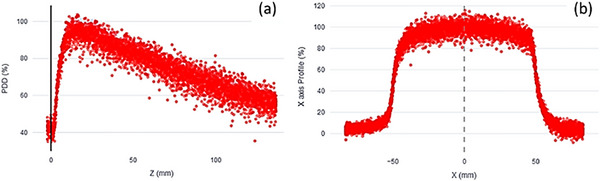
The point cloud corresponding to a PDD (a) and a profile (b) measured by the BP‐PSD for a field size of 10 × 10 cm^2^ at a speed of 10 mm/s.

Ultra‐fast measurements were analyzed using the BP software. As previously mentioned, the BP‐PSD measures the radiation pulse‐by‐pulse and represents it in real time. The BP software displays the measured points corresponding to the signal registered in each pulse as shown in Figure 2. These dots or pulses are indicative of the actual radiation emitted by the linac. The fluctuation observed with respect to the data shown in Figure 2 is partly because the linear accelerator does not emit all pulses equally, although it ultimately adapts the charge per pulse to deliver the correct dose. In contrast, conventional detectors integrate dose over time at discrete positions. As previously stated, the exposure time at each position has a general effect on the statistics of the measurement, since a longer exposure can reduce the uncertainty in the measurement. Given that a large number of pulses are measured per mm, to perform a comparison with the data measured using the Semiflex 3D IC or the micro‐Diamond detector, the BP data were grouped every 20 values and a rolling window interpolation method^53^, ^54^ with mean window size value of 30 was applied to the BP‐PSD data in order to obtain an envelope of all the pulses measured, generating continuous curves. This corresponds to a PDD or profile, depending on the BP‐PSD movements relative to the field axis. The BP software allows the user to select the degree of interpolation and the quantity of shift between the curves. As the BP‐PSD moves, several measures per mm are acquired. The values selected for group size and rolling window size influence the shape of the PDD and profile curves, as well as data as D_max_ for PDDs or penumbra for profiles, and hence the comparison analysis. As previously mentioned, the group size function groups values according to a specified value. The software displays the mean value and the median position of the group. In contrast, rolling window replaces each measurement value with the average of a set of values surrounding that measurement for a specified window size. Rolling window offers the advantage of maintaining the same number of points as the original, while group size ends up with fewer measurement points than the original data. In order to perform the data comparison, values are grouped initially to reduce the quantity of data. Then, the rolling window is applied, resulting in the final curve. The values proposed in this work were determined through a rigorous testing process to ensure optimal performance for all measured curves. However, should further refinement be required, modifications can be made to ensure the values meet the specified criteria.

The sensor uncertainty and noise detector have also been calculated and displayed. Sensor uncertainty was calculated as the standard deviation of signal in the absence of radiation, divided by the square root of the number of measurements in each defined group. It is important to note that these measurements refer to the ones registered by the BP‐PSD between pulses. Sensor noise was computed as the standard deviation of signals within each group during acquisition.

Measured PDD and profiles included in Table 1 were compared to those measured during the commissioning process. Specifically, the PDD were compared with measurements taken using the Semiflex 3D IC and the micro‐Diamond detector, and the profiles were compared with those measured with the micro‐Diamond detector. The data from these detectors were previously acquired point by point during commissioning using the Mephysto software.

To evaluate the differences between curves, a gamma (γ) method analysis^55^ was used within the BP software. The acceptance criterion for dose difference and distance‐to‐agreement (DTA) of 2%/2 mm as proposed by the AAPM^56^ was adopted, although a stricter criterion of 1%/1 mm was also evaluated. PDD and profiles measured with the Semiflex 3D IC or micro‐Diamond diode were set as reference, while the PDD and profiles measured with the BP‐PSD were set as the evaluation distribution. Global dose normalization, γ calculation and a dose threshold of 10% for PDD and 1% for profiles in order to study the out‐of‐field region were applied, following the AAPM TG 218 report recommendations.^57^ The BP software also reported total acquisition time, as well as the point of maximum dose on the PDD (D_max_) and the penumbra data extracted from the profile measurements. Specifically, gamma analysis was performed for PDDs using the measurements taken with both the Semiflex 3D and the micro‐Diamond as the reference dataset. Nevertheless, for profiles, only the measurements obtained with the micro‐Diamond were used as reference for gamma comparison.

## 3 RESULTS

### 3.1 Percentage depth doses

The gamma analysis results for the PDD measurements acquired at scanning speeds of 10 mm/s and 20 mm/s using the BP‐PSD are presented in Table 2. The total acquisition times were 20 s at 10 mm/s and 10 s at 20 mm/s. In comparison, the estimated acquisition time using the micro‐Diamond or Semiflex 3D detectors, as indicated by the Mephysto software, was approximately 144 s, representing a measurement duration between 7 and 14 times longer than that required by the BP‐PSD. The γ analysis showed excellent agreement between the PDD acquired with the BP‐PSD and those acquired during conventional commissioning. When compared with the Semiflex 3D IC, the γ analysis demonstrated 100% agreement under both 2%/2 mm and 1%/1 mm criteria. Comparison with the micro‐Diamond detector also yielded 100% agreement for the 2%/2 mm criterion, and a consistent agreement of 96% for the more stringent 1%/1 mm criterion. The number of data points acquired per millimeter by the BP‐PSD was also analyzed. At a scanning speed of 10 mm/s, the detector registered an average of 169 measurement points and 31 pulses per millimeter. At 20 mm/s, the corresponding values were 71 measurements and 13 pulses per millimeter.

**TABLE 2:** Gamma index for PDD measured with the BP‐PSD compared with the PDD measured with the micro‐Diamond detector and the Semiflex 3D IC.

Field Size (cm^2^)	BP Speed (mm/s)	Ref. detector	Gamma index 2%/2 mm	Gamma index 1%/1 mm
10 × 10	10 mm/s	micro‐Diamond	100	98.44
		Semiflex 3D	100	100
	20 mm/s	micro‐Diamond	100	98.41
		Semiflex 3D	100	100
5 × 5	10 mm/s	micro‐Diamond	100	96.97
		Semiflex 3D	100	100
	20 mm/s	micro‐Diamond	100	96.97
		Semiflex 3D	100	100
3 × 3	10 mm/s	micro‐Diamond	100	100
		Semiflex 3D	100	100
	20 mm/s	micro‐Diamond	100	100
		Semiflex 3D	100	100
2 × 2	10 mm/s	micro‐Diamond	100	100
		Semiflex 3D	100	100
	20 mm/s	micro‐Diamond	100	100
		Semiflex 3D	100	100
1 × 1	10 mm/s	micro‐Diamond	100	100
		Semiflex 3D	100	100
	20 mm/s	micro‐Diamond	100	100
		Semiflex 3D	100	100

Table 3 shows the comparison of the Dmax values registered with the BP‐PSD after the fitting of the curve with the reference. Differences with respect the indicated reference are below 1 mm, although in some field sizes and speeds, the discrepancy in measurements can reach 2 millimeters.

**TABLE 3:** Maximum dose depth (D_max_) values. D_max_ values for Mephysto correspond to those obtained point by point at fixed speed during the reference commissioning procedure and depend on the field size and detector used. The BP D_max_ values correspond to the values obtained after curve fitting with the specified reference detector.

Field Size (cm^2^)	BP speed (mm/s)	BP D_max_ (mm)	Ref. detector	D_max_ mephisto (mm)	Diff. [BP—Ref] (mm)
10 × 10	10 mm/s	13.00	micro‐Diamond	13.80	−0.80
	20 mm/s	13.89			0.09
	10 mm/s	13.50	Semiflex 3D	14.00	−0.50
	20 mm/s	12.74			−1.26
5 × 5	10 mm/s	13.53	micro‐Diamond	13.90	−0.37
	20 mm/s	16.04			2.14
	10 mm/s	12.66	Semiflex 3D	13.60	−0.94
	20 mm/s	13.77			0.17
3 × 3	10 mm/s	13.00	micro‐Diamond	13.30	−0.30
	20 mm/s	14.05			0.75
	10 mm/s	13.27	Semiflex 3D	13.30	−0.03
	20 mm/s	13.83			0.53
2 × 2	10 mm/s	14.07	micro‐Diamond	12.20	1.87
	20 mm/s	12.41			0.21
	10 mm/s	15.79	Semiflex 3D	12.90	2.89
	20 mm/s	13.29			0.39
1 × 1	10 mm/s	13.06	micro‐Diamond	12.00	1.06
	20 mm/s	13.15			1.15
	10 mm/s	13.06	Semiflex 3D	11.60	1.46
	20 mm/s	12.79			1.19

**FIGURE 3:**
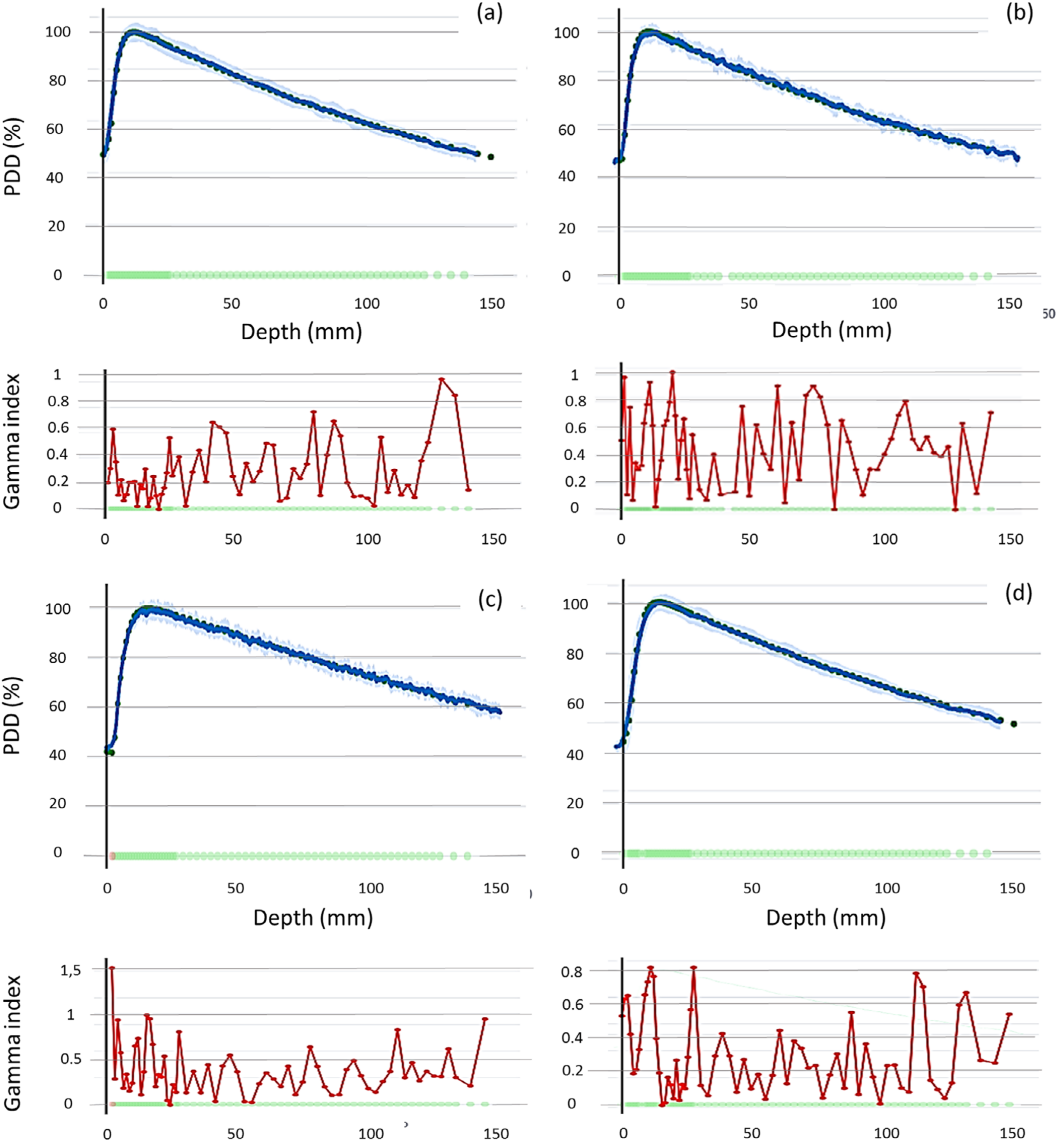
Some PDD comparisons and gamma index between the BP‐PSD and the following field sizes and reference detectors: (a) 1 × 1 cm^2^, 10 mm/s, Semiflex 3D. (b) 1 × 1 cm^2^, 20 mm/s, micro‐Diamond. (c) 10 × 10 cm^2^, 10 mm/s micro‐Diamond. (d) 3 × 3 cm^2^, 20 mm/s, Semiflex 3D. The dots in each graph represent PDD measurements from the reference detector (micro‐Diamond or Semiflex 3D), and the solid line shows the corresponding BP‐PSD measurement.

Figure 3 shows a series of PDD comparisons for various field sizes and BP‐PSD movement speeds. In each PDD comparison, the upper section displays PDD measured with the BP‐PSD alongside the reference PDD acquired using either the micro‐Diamond or Semiflex 3D detector as reference. A blue band represents the radiation noise measured by the BP‐PSD. On the X‐axis, the green dots indicate points that meet the γ criterion, whereas red dots denote points that fail to meet it. The largest discrepancies between the PDD curves occur in the build‐up region, particularly near the surface of the water phantom.

### 3.2 Dose profiles

Table 4 summarizes the results of the γ analysis for profile measurements comparing the BP‐PSD with the micro‐Diamond detector as reference. All measurements met the 2%/2 mm γ passing criterion with 100% agreement, regardless of BP‐PSD scanning speed. For the more stringent 1%/1 mm criterion, superior results were obtained at 10 mm/s, particularly for smaller field sizes (1 × 1 cm^2^ and 2 × 2 cm^2^), with passing rates exceeding 95%. At 20 mm/s, the average γ passing rate across all field sizes remained above 92%, with the lowest individual passing rate still exceeding 80%. For a 10×10 cm^2^ field, the acquisition time using the BP‐PSD was 5 to 9 times shorter than the duration required when using the micro‐Diamond detector, for scanning speeds of 10 mm/s and 20 mm/s, respectively.

**TABLE 4:** Gamma index analysis for dose profiles measured with the BP‐PSD compared with those obtained using the micro‐Diamond detector as reference. Metrics include field size, penumbra width at isocenter (100 mm depth), and acquisition time for each method (BP‐PSD vs Mephysto software).

Field size (cm^2^)	Speed (mm/s)	Direction	Depth (mm)	Gamma index passing rate			Bluephysics PSD					Micro‐diamond detector (mephysto)			
				2%/2 mm	1%/1 mm		Field size (mm)	pen left (mm)	pen. right (mm)	time (s)		Field size (mm)	pen. left (mm)	pen. Right (mm)	time (s)
**10 × 10**	10	cross‐plane	100	100	96.39		100.26	8.82	8.82	29		99.98	7	7	152
		in‐plane	100	100	97.92		99.35	7.05	7.11	28		99.43	6	6	
		cross‐plane	50	100	98.70			8.11	8.22	29					
		in‐plane	50	100	100			6.37	6.52	28					
		cross‐plane	13	100	97.94			6.93	7.09	30					
		in‐plane	13	100	96.39			5.54	5.80	28					
	20	cross‐plane	100	100	83.13		99.79	9.25	9.57	17					
		in‐plane	100	100	93.98		99.36	7.30	7.65	15					
		cross‐plane	50	100	97.40			7.77	7.88	17					
		in‐plane	50	100	81.93			7.42	7.54	15					
		cross‐plane	13	100	81.44			7.54	7.35	17					
		in‐plane	13	100	92.78			6.09	5.88	15					
**5 × 5**	10	cross‐plane	100	100	100		50.37	6.78	7.86	24		49.88	7	7	121
		in‐plane	100	100	100		49.91	6.08	6.24	22		50.14	5	5	
		cross‐plane	50	100	96.10			6.54	7.25	24					
		in‐plane	50	100	97.4			5.55	5.69	22					
		cross‐plane	13	100	87.01			5.97	5.97	24					
		in‐plane	13	100	92.21			5.02	4.93	22					
	20	cross‐plane	100	100	97.40		50.37	7.01	7.81	14					
		in‐plane	100	100	98.70		49.22	6.85	6.85	12					
		cross‐plane	50	100	88.31			6.09	7.10	14					
		in‐plane	50	100	93.51			5.27	5.48	12					
		cross‐plane	13	100	87.01			5.80	5.22	12					
		in‐plane	13	100	85.71			5.29	5.36	12					
**3 × 3**	10	cross‐plane	100	100	89.86		30.39	6.58	7.11	24		29.79	5	6	95
		in‐plane	100	100	98.55		30.07	5.78	5.78	22		29.92	4	4	
		cross‐plane	50	100	100			5.70	6.47	24					
		in‐plane	50	100	97.1			5.45	5.33	22					
		cross‐plane	13	100	95.65			5.50	6.19	24					
		in‐plane	13	100	97.10			4.80	4.80	22					
	20	cross‐plane	100	100	92.75		30.37	6.26	7.07	14					
		in‐plane	100	100	78.26		30.02	5.80	5.91	13					
		cross‐plane	50	100	98.55			5.84	6.22	14					
		in‐plane	50	100	88.41			4.96	4.96	13					
		cross‐plane	13	100	82.61			5.46	6.30	14					
		in‐plane	13	100	91.30			4.87	4.98	12					
**2 × 2**	10	cross‐plane	100	100	100		20.41	5.78	6.78	24		19.77	6	6	82
		in‐plane	100	100	100		20.26	4.80	4.62	22		20.14	4	4	
		cross‐plane	50	100	100			5.39	6.47	24					
		in‐plane	50	100	100			4.53	4.53	22					
		cross‐plane	13	100	100			4.88	5.78	24					
		in‐plane	13	100	100			4.22	4.12	22					
	20	cross‐plane	100	100	98.36		20.43	5.45	6.84	14					
		in‐plane	100	100	93.44		20.05	5.45	5.33	12					
		cross‐plane	50	100	96.72			5.27	6.22	14					
		in‐plane	50	100	98.36			5.22	5.22	12					
		cross‐plane	13	100	93.44			4.87	5.68	14					
		in‐plane	13	100	100			4.75	4.64	12					
**1 × 1**	10	cross‐plane	100	100	100		10.78	4.16	5.70	24		9.78	4	6	68
		in‐plane	100	100	100		10.31	4.27	4.09	22		9.99	4	4	
		cross‐plane	50	100	100			4.09	5.51	24					
		in‐plane	50	100	100			3.78	3.95	22					
		cross‐plane	13	100	100			3.62	5.29	24					
		in‐plane	13	100	100			3.55	3.73	22					
	20	cross‐plane	100	100	96.08		10.93	6.40	3.96	14					
		in‐plane	100	100	98.04		9.94	4.26	4.46	12					
		cross‐plane	50	100	98.04			4.33	5.46	14					
		in‐plane	50	100	96.08			4.06	4.29	13					
		cross‐plane	13	100	98.04			3.96	5.09	14					
		in‐plane	13	100	100			3.45	3.65	13					

Field size discrepancies between the BP‐PSD and micro‐Diamond measurements were most pronounced in the cross‐plane direction and for the smallest field sizes. The maximum observed deviation was 1.15 mm, measured at a scanning speed of 20 mm/s for a 1 × 1 cm^2^ field size. All other field size differences remained below 1 mm, with better agreement consistently achieved in the in‐plane direction. Importantly, all field size measurements remained within ± 1 mm of the nominal values. The penumbra measured using the BP‐PSD was generally broader than that measured with the micro‐Diamond detector, particularly for the largest field size (10 × 10 cm^2^). The smallest differences in penumbra width were observed for the 1 × 1 cm^2^ and 2 × 2 cm^2^ fields at 10 mm/s scanning speed.

For the 10 × 10 cm^2^ field size, the BP‐PSD reduced measurement times by a factor of 5 at 10 mm/s and by nearly a factor of 9 at 20 mm/s, relative to the micro‐Diamond detector. As the field size is reduced to 1 × 1 cm^2^, these reductions were slightly lower, approximately 3 and 5 times faster at 10 mm/s and 20 mm/s, respectively. However, it should be noted that the measurement limits with the BP‐PSD were kept fixed for all field sizes, whereas Mephysto automatically adjusts them depending on field size.

**FIGURE 4:**
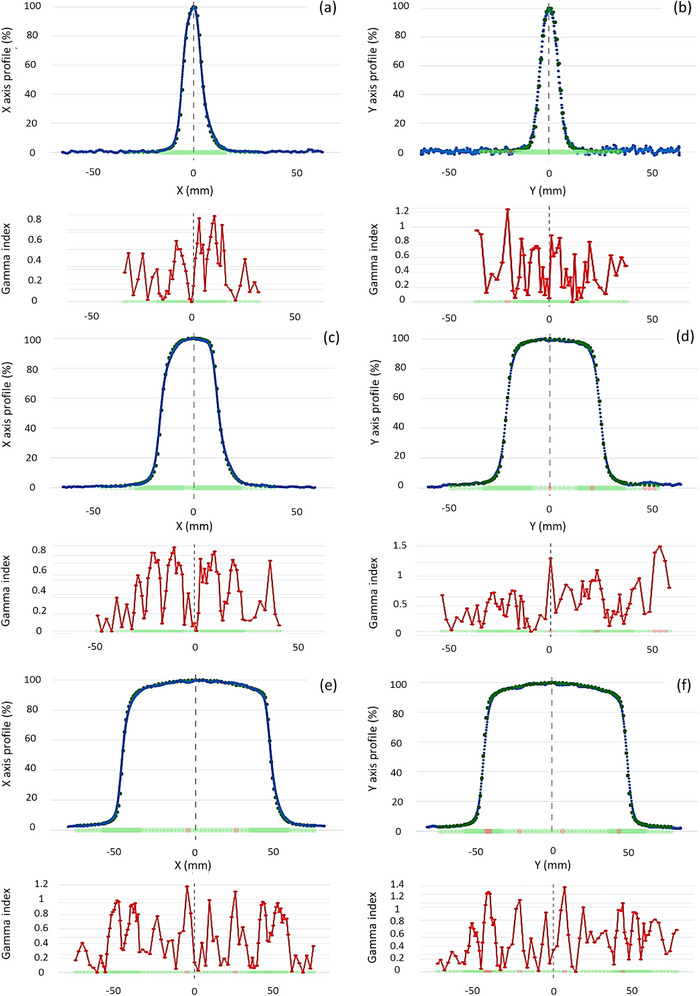
Some comparisons of profiles and γ indices between BP‐PSD and micro‐Diamond (reference) for the following field sizes, depths, directions, and speeds: (a) 1 × 1 cm^2^, 13 mm, cross‐plane, 10 mm/s. (b) 1 × 1 cm^2^, 100 mm, in‐plane, 20 mm/s. (c) 3 × 3 cm^2^, 50 mm, cross‐plane, 10 mm/s. (d) 5 × 5 cm^2^, 50 mm, in‐plane, 20 mm/s. (e) 10 × 10 cm^2^, 13 mm, cross‐plane, 10 mm/s. (f) 10 × 10 cm^2^, 13 mm, cross‐plane, 20 mm/s. The dots in the graph represent the profile measured using the micro‐Diamond as reference, and the solid line represents the profile measured using the BP‐PSD.

Figure 4 shows several examples of profile comparisons between the BP‐PSD and the micro‐Diamond, along with the 1%/1 mm gamma index criterion. As shown in Figure 4, the effect on the measures of the wave in the water phantom created by the movement of the BP‐PSD when measuring the profile can be observed in Figures 4d,f. However, this does not prevent the 2%/2 mm gamma passing rate from being 100%. As displayed in Figure 3, the green dots on the X‐axis indicate points passing the γ criterion, while the red dots indicate points that fail to meet it. Additionally, the blue band indicates BP‐PSD radiation noise, and the light green line corresponds to sensor uncertainty.

## 4 DISCUSSION

Although this work was conducted using an MR‐linac system, the proposed methodology is applicable to other types of linear accelerators and water phantoms that do not have the limitations of the phantom used in this study. According to the manufacturer, the BP system is compatible with a range of commercial water phantoms, enabling ultra‐fast data acquisition across platforms. The Unity MR‐linac was selected due to two key considerations: firstly, to evaluate the BP‐PSD in the most technically demanding environment available, accounting for the influence of the magnetic field and water phantom constraints, challenges that were mitigated through BP software functionality; and secondly, due to greater availability of the MR‐linac at the time the measurements were conducted.

The BP‐PSD demonstrated the ability to acquire PDD and profile data faster than traditional detectors, with high spatial resolution and low measurement uncertainty. This capability supports the feasibility of performing a complete dosimetric commissioning of a linear accelerator within a few hours, without requiring substantial changes to standard commissioning protocols.^5^

All measurements acquired at 20 mm/s satisfied the 2%/2 mm gamma criteria with 100% agreement. However, better compliance with the more stringent 1%/1 mm criterion was observed at 10 mm/s, where the effect of water surface disturbance from detector movement was negligible. Improved performance for smaller fields at higher speeds may be attributed to reduced susceptibility to water disturbance caused by detector movement. This ripple effect on the water surface becomes more prominent in larger fields and at higher speeds, introducing a small, measurable ‘jump’ in the acquired profile, which can be noticed in Figure 4d,f. At 10 mm/s, the movement effect on the water surface is negligible and has no discernible impact on data accuracy. Therefore, it should not be a cause for concern. Measurements taken at 20 mm/s, despite being faster, show this ripple effect more clearly than measurements taken at 10 mm/s, especially when increasing the field size. Therefore, if spatial accuracy is prioritized over acquisition time, it is preferable to take measurements at 10 mm/s, which is still a speed that allows the commissioning of a linac to be completed in just a few hours. The effect of the magnetic field on the shape of the cross‐plane profiles is measured correctly with the BP‐PSD, as illustrated in Figure 4a,c,e.

In summary, while measurements at 20 mm/s were acquired in roughly half the time, 10 mm/s is preferable when higher accuracy is required. In both cases, BP‐PSD significantly outperformed IC and diodes in terms of acquisition speed. Specifically, ultra‐fast BP‐PSD measurements offer significant advantages in terms of time saving. In the case of an MR‐linac, with the water phantom that only allows point‐to‐point measurements, they are up to 5 and 7 times faster for PDD and profiles, respectively, and a 10 × 10 cm^2^ field. These ultra‐fast measurements would allow the dosimetric commissioning of both an MR‐linac and a conventional one in a few hours, which can be highly advantageous in busy centers that receive multiple linacs at the same time.

It is important to note that the sampling strategies differ substantially between the BP‐PSD and traditional detectors. Standard systems such as the Semiflex 3D or micro‐Diamond acquire point‐by‐point measurements every 1–2.5 mm, pausing at each position for data collection. By contrast, the BP‐PSD captures 169 and 71 data points per mm at 10 mm/s and 20 mm/s, respectively, yielding much denser and more continuous datasets. This extensive sampling may be further optimized using the software's built‐in grouping functionality to enhance signal interpretation. Care must be taken when selecting the parameters of the rolling window technique, as both the window size and the data points per window affect the results. In this study, it was observed that a group and window size of 20 and 30, respectively, yielded good results without generating excessively noisy or smoothed curves. Nevertheless, the observed differences in D_max_ between BP‐PSD and micro‐Diamond or Semiflex 3D detectors are likely attributable to the selection of these parameters for the complete PDD curves and the detector positioning rather than to the BP‐PSD response characteristics. As shown in Table 3, these differences exceed the 1 mm tolerance established in various protocols^1^, ^5^, ^58^ for the D_max_ value or detector positioning for some field sizes and speeds. The same reasoning can be applied to the measured profiles. The selected parameters for the group and window size are common to all profile regions, the central region, the penumbra, and the umbra. In order to achieve an optimal match, the penumbra was slightly broadened. This is more clearly noticeable in larger fields at higher speeds. Similar results were obtained in previous works^47^, ^48^ although point by point measures with the BP‐PSD showed better results in terms of penumbra, which was also slightly greater with BP‐PSD than with micro‐Diamond. This suggests that the finite size of the scintillator or the coating within which it is enclosed may also influence the measurement outcomes.

A potential future enhancement to the BP software could involve the introduction of a feature that allows users to select parameters based on the specific region of the curve that requires manipulation, applicable to both PDD and profiles. Previous studies have undertaken measures point by point with this same BP‐PSD and a MR‐linac^47^, ^49^ or in continuous mode^48^ with an C‐arm type linac and standard velocity. These studies measured profiles, PDD or output factors and obtained excellent results when compared with other detectors; consequently, output factors measures were not undertaken in the present study.

Although deep‐learning‐based methods continue to improve in modelling beam data and generating synthetic PDD and profiles^59^ for potential commissioning and QA applications^36^, ^37^ physical ultra‐fast measurements with the BP‐PSD remain advantageous in terms of both speed and direct insight into beam behavior. Furthermore, given the importance of the commissioning in establishing a reference baseline for linac performance, it seems more appropriate to perform the physical measurements rather than relying on AI methods or machine golden data.^32^, ^35^

While PDD and profile measurements required for commissioning a linac with BP‐PSD can be completed in less time than with other detectors, some measurements with the latter^5^, ^38^, ^41^ would still be necessary to ensure the adjustment. The use of this technology for ultra‐fast dosimetric commissioning of a linac is still in its early stages. It is recommended that the linac be commissioned with multiple detectors^5^ so that a complete dosimetric commissioning could be carried out with the BP‐PSD, followed by measurements with other detectors to verify the results.

The BP software could be improved to fully automate the entire adjustment process. Ultimately, all the effort to smooth the measurements based on the original pulse signals is only intended to match the original and historical data taken with conventional detectors such as IC with much lower temporal resolution. The BP‐PSD provides accurate information on how the linac behaves by directly displaying the measurement of each radiated pulse. This presents a new opportunity to enhance the quality control of the accelerator and prevent future failures, as has been previously proposed in some studies.^60^

For future work, it would be ideal to obtain the original commissioning data using high temporal resolution detectors. This would allow direct comparison of accelerator pulses during annual checks without the need to smooth the data. Statistical methods could then be applied to verify that pulses measured during commissioning are within tolerance with those from subsequent checks. By using the BP‐PSD for both initial and follow‐up measurements, comparisons could be made without relying on other detectors like ICs or diodes. This approach offers a promising direction for future research and software development.

## 5 CONCLUSION

The BP‐PSD is capable of acquiring PDD and dose profiles with high accuracy and significantly faster than conventional detectors. This is particularly advantageous when commissioning a linear accelerator in a time‐constrained environment, ensuring the same level of precision and reliability as traditional methods with IC or diode‐based systems. The direct connection of the BP‐PSD system to any water phantom represents a significant advantage in terms of convenience. It allows all measurements to be programmed, with the software performing the movements at the specified speed in a short time.

Additionally, the BP‐PSD pulse‐by‐pulse measuring mode can be utilized for small field dosimetry or output factors measurement. Subsequent research may explore methods of performing not only ultra‐fast commissioning, but also subsequent linac QA by means of direct data analysis. This analysis would be based on the measured pulses, which indicate what the machine has actually radiated.

## AUTHOR CONTRIBUTIONS

All authors participated in data collection, analysis, writing and editing of the manuscript.

## CONFLICT OF INTEREST STATEMENT

M. Feijoo and A. Cardin are employees of the Blue Physics PSD system.
